# Comparison of Post‐Cementation Sensitivity Between Resin Modified Glass Ionomer Cement and Conventional Glass Ionomer Cement as a Luting Material—A Randomized Clinical Trial

**DOI:** 10.1002/cre2.70339

**Published:** 2026-03-31

**Authors:** Shamsul Alam, Naveed Sadiq, Hend Mohamed Elsayed, Sheema Shakir, Nasser Hussein Shaheen, Ahmed Mohammed Elmarakby, Asif Rehman

**Affiliations:** ^1^ Department of Public Health Khyber Medical University Peshawar Pakistan; ^2^ Institute of Public Health and Social Sciences Khyber Medical University Peshawar Pakistan; ^3^ Restorative Dentistry, Department of Restorative and Prosthetic Dental Sciences, College of Dentistry Dar Al Uloom University Riyadh Saudi Arabia; ^4^ Restorative Dentistry, Department of Restorative and Prosthetic Dental Sciences, College of Dentistry Cairo University Giza Egypt; ^5^ Department of Prosthodontics Khyber College of Dentistry Peshawar Pakistan; ^6^ Restorative and Prosthetic Dental Sciences Department Dar Al Uloom University Riyadh Saudi Arabia; ^7^ Department of Operative Dentistry, Faculty of Dental Medicine Al Azhar University, Assiut Branch Cairo Egypt; ^8^ Department of Restorative and Prosthetic Dental Sciences, College of Dentistry Dar Al Uloom University Riyadh Saudi Arabia; ^9^ Bath Spa University Bath UK

**Keywords:** fixed partial denture, post‐cementation sensitivity, resin‐modified glass ionomer cement

## Abstract

**Objective:**

To compare post‐cementation sensitivity between resin‐modified glass ionomer cement (RMGIC) and conventional glass ionomer cement (GIC) used as luting agents for single crowns.

**Materials and Methods:**

This randomized controlled trial was conducted at Saidu College of Dentistry, Swat, Pakistan. A total of 496 eligible participants were randomized into two groups: GIC (*n* = 248) and RMGIC (*n* = 248). Post‐cementation sensitivity was recorded at 24 h, 1 week, and 1 month using a 10‐point visual analog scale (VAS), with scores categorized as no, mild, moderate, or severe sensitivity. Data were analyzed using Mann–Whitney *U* tests, and linear mixed‐effects models adjusted for age, gender, jaw, and prosthesis location.

**Results:**

Baseline demographic and clinical characteristics were comparable between groups. Post‐cementation sensitivity was consistently lower in the RMGIC group compared to the GIC group across all time points. Median sensitivity scores at 24 h were 4.0 (GIC) versus 3.0 (RMGIC, *p* < 0.001); at 1 week, 2.0 versus 1.0 (*p* < 0.001); and at 1 month, both minimal but significantly lower in the RMGIC group (*p* < 0.001). The adjusted mixed‐effects model confirmed that RMGIC significantly reduced sensitivity compared to GIC (*β* = −0.53; OR 0.59, 95% CI 0.46–0.75; *p* < 0.001). Sensitivity was highest at 24 h (*β* = 2.96; OR 19.3, 95% CI 15.3–24.3; *p* < 0.001), followed by 1 week (*β* = 0.81; OR 2.25, 95% CI 1.77–2.86; *p* < 0.001), and declined markedly by 1 month. Age showed a modest protective effect (*β* = −0.008; OR 0.99, 95% CI 0.99–1.00; *p* = 0.04), while gender, jaw, and prosthesis location were not significant predictors. A significant interaction at 24 h showed RMGIC provided an additional reduction in sensitivity compared to GIC (*β* = −0.83; OR 0.44, 95% CI 0.31–0.61; *p* < 0.001).

**Conclusion:**

RMGIC demonstrated significantly lower post‐cementation sensitivity compared to conventional GIC.

**Trial Registration:**

This trial was retrospectively registered with Clinical Trials Registry of ClinicalTrials.gov(registration#NCT07102121) on August 3, 2025.

**Abbreviations**
CI: confidence interval
FDP: fixed partial denture
GIC: glass ionomer cement
OR: odds ratio
RCT: randomized controlled trial
RMGIC: resin‐modified glass ionomer cement
VAS: visual analog scale

## 1 Introduction

Dental prostheses are commonly required to restore the missing teeth (Kern et al. 2017). Single full‐coverage crowns are a standard treatment option to rehabilitate function, phonetics, and esthetics of damaged or extensively restored teeth (Attia et al. 2019). These crowns require retention and sealing to prepared tooth surfaces using materials known as luting cements (Agha et al. 2020). The ideal requirements of these luting materials are a lack of solubility, good compatibility with the host, no leakage, and no post‐cementation pain or sensitivity (Abad‐Coronel et al. 2019). The luting materials are glass ionomer cement (GIC), resin‐modified glass ionomer cement (RMGIC), zinc phosphate cement, and zinc oxide eugenol cement. GIC and RMGIC are considered durable materials for luting purpose (Bakhadher 2019; Almuhaiza 2016).

For newly cemented fixed dental prosthesis postoperative sensitivity is a common complication (Suzuki 2000). Postoperative sensitivity is sharp pain of short duration to thermal stimuli in patients wearing a newly cemented crown (Iqbal et al. 2018). Crown preparation culminates in the exposure of dentinal tubules and activating pain nerve endings of the pulp. Most of the luting agents have acidic ingredient which produce chemical stimuli and results in post‐cementation sensitivity (Gupta et al. 2013).

Glass ionomer cement is commonly used for luting, but it has relatively high solubility. To combat this issue, RMGIC has been developed and has less solubility as compared to GIC due to the incorporation of resin. A randomized clinical trial was conducted in India on 50 patients, in which half crowns were cemented with GIC and half with RMGIC. Their results showed that post cementation pain at day 7 was higher in GIC than RGMIC statistically (*p* < 0.05) (Mehta et al. 2012). Another study reported that post‐cementation sensitivity in 13.3% of subjects with resin cement and in only 5.9% of subjects with GIC (Denner et al. 2007). This study is being conducted to determine which material causes less post‐cementation sensitivity. Post‐cementation sensitivity can be most disturbing for patients and may affect the quality of life.

Post‐cementation sensitivity is a subjective outcome that may vary among different populations due to genetic, ethnic, and biological factors. The present study addresses this variability by evaluating whether a RMGIC, which is considered more durable, provides outcomes equivalent to or better than a conventional GIC in terms of post‐cementation sensitivity. Therefore, this study was conducted to compare post‐cementation sensitivity between RMGIC and conventional GIC when used as a luting agent.

## 2 Material and Methods

### 2.1 Trial Design, Ethical Considerations, and Registration

This double‐blind, prospective, parallel randomized controlled trial was conducted at the Department of Prosthodontics, Saidu College of Dentistry, Swat, Pakistan, from 25th January 2025 to 3rd September 2025. A non‐probability sampling technique was used, whereby all patients who met the inclusion criteria and presented during the study period were enrolled without omission. This approach is commonly applied in hospital‐based research where the source population is not fully defined. Ethical approval was obtained from the hospital's ethical committee (8643/SCD/Swat/Ethical/Certificate). Written informed consent was obtained from patients or their guardians (for participants under 18 years) as per the Helsinki declaration. Participants were assured that their personal and clinical information would remain confidential. This trial was registered with ClinicalTrials.gov (NCT07102121) on August 3, 2025. The study was conducted in accordance with the CONSORT 2025 guidelines for randomized trials (Hopewell et al. 2025).

### 2.2 Sample Size

The total sample size was 496 participants (248 in each group), calculated using post‐cementation sensitivity rates of 13.3% for RMGIC and 5.9% for GIC reported in a previous study (Denner et al. 2007) with a 5% level of significance and 80% power, through OpenEpi software.

### 2.3 Participants

**Figure 1:**
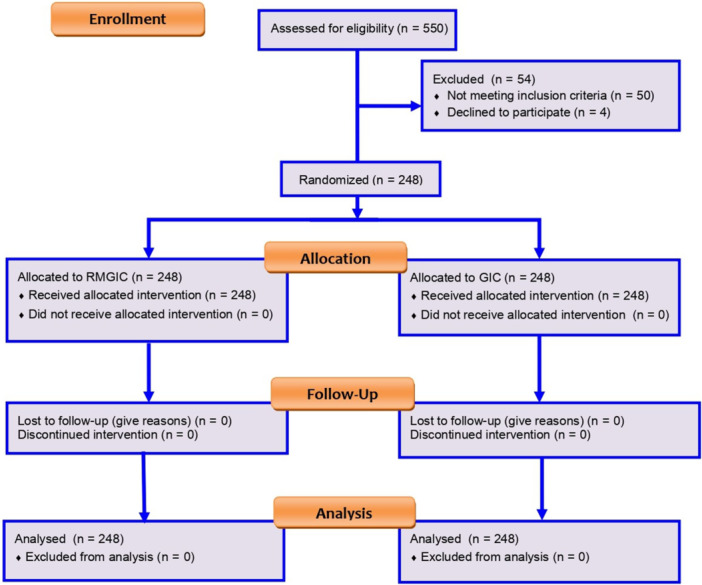
CONSORT flow diagram.

A total of 550 patients scheduled for fixed prosthodontic treatment were screened according to the inclusion criteria. Details are provided in Figure 1.

Participants aged 15–70 years, of either gender, requiring a single crown on anterior (incisors and canines) or posterior (premolars and molars) teeth, with abutment teeth exhibiting vital pulps and sound structure without signs of attrition, erosion, or abrasion, were included. Only teeth without a history of pulp capping were included. Patients were excluded if they were undergoing orthodontic treatment or had completed it within the previous 3 months, had a history of periodontal surgery, or were using bleaching or desensitizing agents. Individuals taking medications that could influence pulpal or periodontal response, such as analgesics or carbamazepine, were also excluded, as were psychiatric patients.

### 2.4 Randomization, Allocation, and Blinding

A list of 496 eligible patients was prepared from the departmental appointment diary, and each patient was assigned a unique identification number. The participants were then randomly allocated into two groups (Group A: receiving cementation with conventional GIC; Group B: receiving cementation with RMGIC) using a simple randomization technique. Random numbers were generated in Microsoft Excel 2016 using the RAND() function and applied to the identification numbers. Randomization was carried out by an independent researcher who was not involved in patient recruitment or treatment. To ensure allocation concealment, the random sequence was secured in sequentially numbered, opaque, sealed envelopes, which were opened only at the time of cementation. Both the patients and the outcome‐recording researcher were blinded. The patients were unaware of the type of cement used, and all cementation procedures were performed by operators other than the primary researcher.

### 2.5 Interventions

At the time of cementation, isolation was achieved using cotton rolls. The abutment teeth were dried with an air spray before cementation. Manufacturer's instructions (GC Corp., Japan) were strictly followed for the mixing and application of the luting materials to the crowns. For conventional GIC, the powder and liquid were dispensed in a ratio of 1 scoop of powder to 1 drop of liquid and mixed on a glass slab for approximately 30–45 s until a homogeneous consistency was achieved. For RMGIC, the material was dispensed in the recommended ratio of 1 scoop of powder to 1 drop of liquid and mixed within 30 s. The mixed cement was applied to the internal surface of the crown, and cementation was carried out with firm finger pressure. In the RMGIC group, the material was additionally light‐cured for 10 s using an LED curing unit, in accordance with the manufacturer's guidelines. Prior to cementation, occlusal adjustment was performed in all cases to eliminate premature contacts. Isolation was maintained for 10 min after completion of the procedure. The patients were instructed not to eat for 1 h, to avoid a hard diet for 24 h, and to refrain from taking any pain‐control medications during the study period. Patients were subsequently contacted by telephone and asked to report the presence of post‐cementation sensitivity at 24 h, 1 week, and 1 month.

### 2.6 Outcome Measurement

Post‐cementation sensitivity was recorded using a 10‐point visual analog scale (VAS), where a score of 0 indicated no pain or sensitivity and a score of 10 represented the worst possible pain or sensitivity.

### 2.7 Bias

Bias and potential confounders were addressed through prespecified inclusion/exclusion criteria, randomized allocation with allocation concealment (details provided in the Section 2.4), and blinded outcome assessment. To further reduce analytical bias, repeated measures were modeled using a linear mixed‐effects approach with adjustment for relevant covariates (age, gender, jaw, and prosthesis location), thereby minimizing residual confounding in the results.

### 2.8 Statistical Analysis

All data analyses were performed using R software (version 4.3.3). Descriptive statistics were computed for demographic and clinical variables. Mean post‐cementation sensitivity scores were compared between the two groups across follow‐up intervals (24 h, 1 week, and 1 month) using the Mann–Whitney *U* test. Post‐cementation sensitivity was analyzed using a linear mixed‐effects model with a subject‐specific random intercept to account for within‐participant correlation across repeated measurements. Fixed effects included cement type (conventional GIC vs. resin‐modified GIC), time points, and their interaction, with adjustment for age, gender, jaw (mandible vs. maxilla), and prosthesis location (anterior vs. posterior). Results are presented as coefficient estimates with 95% confidence intervals (CI). A *p* value < 0.05 was considered statistically significant.

## 3 Results

Out of 550 patients screened, 496 were eligible and randomized: GIC (*n* = 248) and RMGIC (*n* = 248) (Figure 1).

Baseline characteristics were comparable between groups: mean age (36.5 vs. 37.3 years, *p* = 0.38), gender distribution (53.2% vs. 58.1% male, *p* = 0.28), jaw involvement (59.3% vs. 60.1% mandible, *p* = 0.85), and prosthesis location (61.7% vs. 62.5% posterior, *p* = 0.85) (Table 1).

**Table 1:** Distribution of demographics and location of prostheses of the participants.

Characteristic	GIC *N* = 248	RMGIC *N* = 248	*p*‐value
Age in years	36.54 (9.12)	37.25 (9.02)	0.38*
Gender, *n*(%)			0.28**
Female	116 (46.77)	104 (41.94)	
Male	132 (53.23)	144 (58.06)	
Jaw, *n*(%)			0.85**
Mandible	147 (59.27)	149 (60.08)	
Maxilla	101 (40.73)	99 (39.92)	
Location of prosthesis, *n*(%)			0.85**
Anterior	95 (38.31)	93 (37.50)	
Posterior	153 (61.69)	155 (62.50)	

*Note:* Mean (SD); *n* (%).

* Welch two sample *t*‐test.

** Pearson's chi‐squared test.

Post‐cementation sensitivity was significantly lower in the RMGIC group compared with the GIC group at all follow‐up points (Table 2). At 24 h, the median sensitivity score was 4.0 (IQR 2.0–6.0) for GIC versus 3.0 (IQR 1.0–4.0) for RMGIC (*p* < 0.001). After 1 week, scores decreased to 2.0 (IQR 1.0–3.0) for GIC and 1.0 (IQR 0.0–2.0) for RMGIC (*p* < 0.001). By 1 month, sensitivity was minimal in both groups but remained significantly lower with RMGIC (median 1.0 [IQR 0.0–1.0] vs. 1.0 [IQR 0.0–2.0]; *p* < 0.001) (Table 2).

**Table 2:** Comparison of post‐cementation sensitivity at various time points between glass ionomer versus resin modified glass ionomer cement.

Post cementation sensitivity	GIC (*n* = 248)	RMGIC (*n* = 248)	*p*‐value*
At 24 h	4.0 (2.0, 6.0)	3.0 (1.0, 4.0)	< 0.001
After 1 week	2.0 (1.0, 3.0)	1.0 (0.0, 2.0)	< 0.001
After 1 month	1.0 (0.0, 2.0)	1.0 (0.0, 1.0)	< 0.001

*Median(IQR); Mann–Whitney *U* test.

In the adjusted linear mixed‐effects model, participants receiving resin‐modified GIC (RMGIC) reported significantly lower post‐cementation sensitivity compared with those receiving conventional GIC (*β* = −0.53, 95% CI −0.77 to −0.29; *p* < 0.001). Sensitivity scores were highest at 24 h (*β* = 2.96, 95% CI 2.73–3.19; *p* < 0.001) and remained elevated at 1 week (*β* = 0.81, 95% CI 0.57–1.05; *p* < 0.001) compared with 1 month. Increasing age was associated with a small reduction in sensitivity (*β* =−0.008 per year, 95% CI −0.015 to −0.000; *p* = 0.04). Gender, jaw (maxilla vs. mandible), and prosthesis location (posterior vs. anterior) were not significantly associated with sensitivity. A significant interaction between cement type and time was observed at 24 h, where RMGIC was associated with a larger reduction in sensitivity compared with GIC (interaction *β* = −0.83, 95% CI −1.16 to −0.50; *p* < 0.001). No significant interaction was seen at 1 week (*p* = 0.79) (Table 3).

**Table 3:** Linear mixed‐effects model of demographic and clinical predictors of post‐cementation sensitivity over time.

Predictor	Estimate (*β*)	95% CI	*p* value*
Cement type			
GIC (reference)	—	—	—
RMGIC	−0.53	−0.77 to −0.29	< 0.001
Time point			
1 month (reference)	—	—	—
1 week	0.81	0.57–1.05	< 0.001
24 h	2.96	2.73–3.19	< 0.001
Age (per year)	−0.008	−0.015 to −0.000	0.04
Gender			
Female (reference)	—	—	—
Male	0.06	−0.08 to 0.20	0.41
Jaw			
Mandible (reference)	—	—	—
Maxilla	−0.06	−0.20 to 0.08	0.41
Prosthesis location			
Anterior (reference)	—	—	—
Posterior	0.06	−0.08 to 0.20	0.40
Cement × time interaction			
RMGIC × 1 week	0.04	−0.29 to 0.38	0.79
RMGIC × 24 h	−0.83	−1.16 to −0.50	< 0.001

*Linear mixed‐effects model.

**Figure 2:**
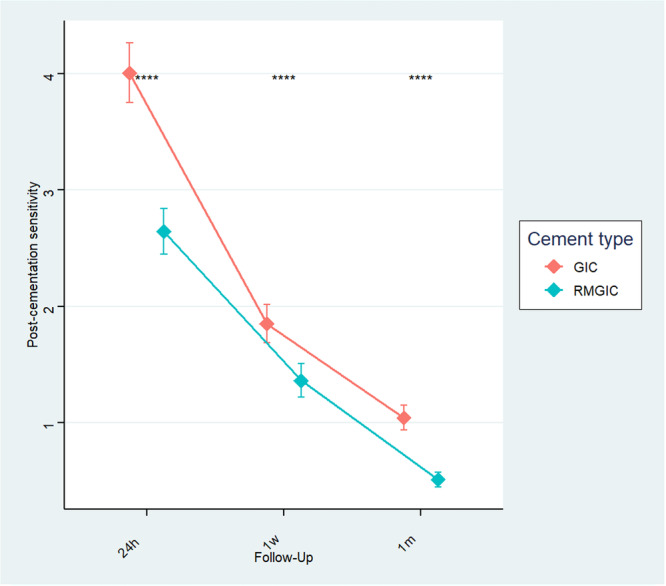
Pattern of post cementation sensitivity in both type cements over time.

Post‐cementation sensitivity was significantly higher in the GIC group compared with the RMGIC group at all follow‐up intervals (24 h, 1 week, and 1 month; *p* < 0.001 for each, paired *t* test). Both groups showed a progressive decline in sensitivity over time, with the steepest reduction occurring within the first week. By 1 month, sensitivity scores were minimal in both groups, though consistently lower in the RMGIC group (Figure 2).

## 4 Discussion

This randomized clinical trial demonstrated that RMGIC resulted in significantly reduced postoperative dentinal hypersensitivity compared with conventional GIC across all evaluated time intervals. The highest sensitivity was observed at 24 h following cementation, with a marked reduction within the first week and minimal symptoms by 1 month.

The clinical advantage of RMGIC can be attributed to its enhanced physical and chemical properties. The incorporation of resin components improves early mechanical strength and significantly reduces solubility in the oral environment compared with conventional GIC. Reduced solubility limits material dissolution and ion release during the early setting phase, thereby decreasing pulpal irritation and postoperative sensitivity. These properties likely explain the more rapid reduction and consistently lower sensitivity scores observed in patients treated with RMGIC (Sidhu 2010; Shenoy et al. 2025).

Baseline demographic and clinical characteristics, including age, sex, jaw distribution, and prosthesis location, were comparable between groups, minimizing the risk of confounding (De Boer et al. 2015). In the adjusted linear mixed‐effects model, the use of RMGIC remained a significant independent predictor of lower post‐cementation sensitivity, underscoring the robustness of the findings. Time was also an important determinant, with peak sensitivity reported at 24 h, followed by substantial improvement within 1 week.

Age demonstrated a modest but statistically significant inverse association with sensitivity. This observation is biologically plausible, as advancing age is accompanied by secondary dentine deposition and progressive dentinal sclerosis, which decrease the diameter and patency of dentinal tubules (Xie et al. 2023). Consequently, fluid movement within the pulp–dentine complex is reduced, thereby diminishing pulpal excitability and the subjective perception of pain (Farag et al. 2025). Additionally, age‐related reductions in pulpal innervation and pulp chamber volume may further attenuate sensory responsiveness (Blatz et al. 2013).

Hilton et al. (2004) conducted a larger multicenter practice‐based trial comparing conventional glass ionomer cement (GIC) with RMGIC for full‐coverage crowns. In contrast to our results, they found no significant differences between the two luting agents at any time point, although overall postoperative sensitivity levels remained low. A likely reason for this variation lies in methodological differences: Hilton et al. relied solely on patient self‐reported outcomes, whereas our study incorporated repeated clinical follow‐up assessments and statistical modeling of time–cement interactions, which may have enhanced the ability to detect subtle differences. Both studies consistently demonstrated an inverse relationship between patient age and postoperative sensitivity, reinforcing the biological plausibility of our age‐related observations.

Shetty et al. (2009) compared conventional GIC with adhesive resin cement and reported significantly greater postoperative sensitivity with GIC at 7 days, although no differences were noted immediately or at 24 h. These findings align with our observation that GIC is associated with more prolonged sensitivity compared to resin‐based cements, although the onset of divergence occurred later in their trial than in ours. Variations in cement composition and differences in assessment intervals may help explain this discrepancy.

Other resin‐based cements, such as the 4‐META–containing adhesive resin cement evaluated by Denner et al. (2007), demonstrated no significant difference from conventional GIC in terms of postoperative sensitivity. In their 2‐year split‐mouth clinical trial, both cements showed only transient hypersensitivity at early follow‐up, which resolved completely by 24 months. These findings suggest that long‐term outcomes are influenced more by material properties and clinical protocols than by cement type alone. A key explanation for early differences is the low initial setting pH of GIC, which has been implicated in postoperative sensitivity (Kamal et al. 2000). Additionally, variations in powder–liquid ratio during manual mixing can compromise the mechanical integrity of the cement, increasing porosity and prolonging setting time. Capsule‐based GICs minimize this variability by ensuring a consistent ratio, whereas resin cements—lacking an acidic liquid component—are not affected by mixing inconsistencies, which may explain their comparable or lower incidence of hypersensitivity (Farag et al. 2025).

### 4.1 Generalizability of the Study

This randomized controlled trial provides reliable results because of its careful design, including clear inclusion and exclusion criteria, proper randomization, blinding, and standardized clinical protocols. However, the extent to which these findings can be generalized should be considered with some caution. Since the study was conducted in a single dental teaching hospital and used a non‐probability sampling method, the participants may not fully reflect patients from different regions, socioeconomic groups, or healthcare settings. Moreover, only patients requiring single crowns on vital teeth without complications such as attrition, erosion, or significant periodontal problems were included, which makes the results more applicable to routine prosthodontic cases rather than complex ones. Still, the relatively large sample size, wide age range (15–70 years), and inclusion of both male and female patients make the findings clinically relevant for general dental practice. Overall, the results are valuable for similar hospital‐based settings, but multicenter studies involving more diverse populations would be needed to confirm broader applicability.

Our follow‐up period was limited to 1 month because post‐cementation sensitivity is largely an acute and self‐limiting response that occurs soon after luting and rarely persists long term. Over the scheduled visits within that month, patients showed a steady reduction in reported sensitivity, reaching almost zero by the end of the observation period. This pattern suggests that the pulp and periodontium adapt within the early healing window and that prolonged monitoring would not meaningfully change the conclusions regarding sensitivity. However, because longer‐term biological behavior and material performance were not evaluated, our findings should be interpreted as reflecting only the short‐term, acute phase response.

### 4.2 Limitations and Future Direction

This study has several important limitations. It was conducted in a single dental teaching hospital using a non‐probability sampling method, which limits the external validity of the findings and may not reflect outcomes in other clinical environments or patient populations. The trial included only patients receiving single crowns on vital teeth without extensive structural loss, prior endodontic treatment, or advanced periodontal compromise; therefore, the applicability of the results is restricted to routine prosthodontic cases rather than complex rehabilitations. The follow‐up duration of 1 month captures only the acute phase and does not allow evaluation of medium‐ or long‐term biological responses to the cements. Additionally, relevant modifying factors such as dietary habits, oral hygiene practices, and occlusal loading were not controlled for, although they may influence post‐cementation sensitivity. Finally, sensitivity assessment was performed via telephone interviews rather than clinical examination, which may introduce response bias and limit diagnostic precision.

Future studies should be conducted in multiple centers with larger and more diverse populations to improve generalizability. Longer follow‐up is needed to evaluate long‐term sensitivity and overall clinical performance of luting cements. Including patients with more complex restorative needs, such as multiple‐unit bridges or compromised abutments, would provide more comprehensive evidence. The use of advanced diagnostic methods, like pulp vitality testing and digital occlusal analysis, could also help clarify the biological and mechanical factors contributing to post‐cementation sensitivity and guide more precise material selection in clinical practice.

## 5 Conclusion

This clinical trial showed that crowns cemented with RMGIC resulted in less post‐cementation sensitivity compared to conventional GIC. The difference was most noticeable within the first 24 h, when sensitivity was at its peak, and remained in favor of RMGIC during the first week and at 1 month. Although both groups showed a natural decline in sensitivity over time, RMGIC consistently provided better patient comfort. Age showed a significant influence on sensitivity, while gender, tooth position, and jaw location had no significant effect. Clinically, these findings support the use of RMGIC as a preferable luting material for crown cementation due to its reduced postoperative sensitivity. Further studies with longer follow‐up and more complex restorative cases are recommended to confirm this outcome.

## Author Contributions

**Shamsul Alam:** conceptualization (equal), data collection (equal), writing – review and editing (equal), final approval. **Naveed Sadiq:** conceptualization (equal), data curation (equal), investigation (equal), writing (equal), supervision (equal). **Hend Mohamed Elsayed:** conceptualization (equal), data analysis (equal), writing – review and editing (equal), final approval. **Sheema Shakir:** conceptualization (equal), data interpretation (equal), writing – review and editing (equal), final approval. **Nasser Hussein Shaheen:** conceptualization (equal), literature review (equal), methodology (equal), writing – review and editing (equal), final approval. **Ahmed Mohammed Elmarakby:** conceptualization (equal), literature review (equal), methodology (equal), writing – review and editing (equal), final approval. **Asif Rehman:** conceptualization (equal), data curation (equal), literature review (equal), data analysis (equal), methodology (equal), writing – review and editing (equal), final approval.

## Funding

The authors received no specific funding for this work.

## Ethics Statement

The study was approved by the hospital committee of Saidu College of Dentistry, Swat, Pakistan (reference number: 8643/SCD/Swat/Ethical/Certificate).

## Consent

Informed written consent was obtained from all participants and/or their guardians in the case of those under the age of 16 years prior to their enrollment in the study. This study does not contain any individual person's data in any form (including images or videos).

## Conflicts of Interest

The authors declare that they have no financial, personal, or professional conflicts of interest that could have influenced the work reported in this manuscript. No support or funding was received from any organization that could be perceived to influence the findings, and there are no affiliations or relationships that may be viewed as potential competing interests.

## Data Availability

Data of current study are available from the corresponding author on reasonable request. However, for privacy reasons, no individual data allowing identification of participants (e.g., videos) can be provided.
